# Hands-Off and Hands-On Casting Consistency of Amputee below Knee Sockets Using Magnetic Resonance Imaging

**DOI:** 10.1155/2013/486146

**Published:** 2013-11-19

**Authors:** Mohammad Reza Safari, Philip Rowe, Angus McFadyen, Arjan Buis

**Affiliations:** ^1^Department of Orthotics and Prosthetics, University of Social Welfare and Rehabilitation Sciences, Tehran 1985713834, Iran; ^2^Department of Biomedical Engineering, University of Strathclyde, Curran Building, 131 St. James Road, Glasgow G4 0LS, UK; ^3^AKM-STATS, Glasgow G1 1EX, UK

## Abstract

Residual limb shape capturing (Casting) consistency has a great influence on the quality of socket fit. Magnetic Resonance Imaging was used to establish a reliable reference grid for intercast and intracast shape and volume consistency of two common casting methods, Hands-off and Hands-on. Residual limbs were cast for twelve people with a unilateral below knee amputation and scanned twice for each casting concept. Subsequently, all four volume images of each amputee were semiautomatically segmented and registered to a common coordinate system using the tibia and then the shape and volume differences were calculated. The results show that both casting methods have intra cast volume consistency and there is no significant volume difference between the two methods. Inter- and intracast mean volume differences were not clinically significant based on the volume of one sock criteria. Neither the Hands-off nor the Hands-on method resulted in a consistent residual limb shape as the coefficient of variation of shape differences was high. The resultant shape of the residual limb in the Hands-off casting was variable but the differences were not clinically significant. For the Hands-on casting, shape differences were equal to the maximum acceptable limit for a poor socket fit.

## 1. Introduction

The purpose of the prosthetic socket is to provide a mechanical coupling between the residual limb and the prosthesis. The overall success of the prosthesis is influenced by the quality of this coupling. Socket fit is the most important characteristic of a prosthesis indicated by amputees [1, 2]. The Patellar Tendon Bearing (PTB) socket was first introduced, by Radcliffe in the 1950s, based on gait biomechanics [3]. According to PTB principles the residual limb is loaded proportionally to the load tolerance of the underlying soft tissue and bony areas. In the 1990s the ICECast casting system was introduced based on hydrostatic principle of load transfer to achieve a uniform pressure distribution [4]. In studies there is a controversy over absolute indication, amputee satisfaction, comfort, performance, and gait for these socket designs [5–10].

Despite many studies on different aspects of transtibial sockets and residual limbs, there is a lack of knowledge to enable consistent manufacturing of a comfortable socket and desirable alignment without the need for several trial and error fittings [11]. The socket is usually made through the process of shape capturing, rectification, and alignment. Before any comparison can be made, repeatability in the socket shape and volume is the fundamental factor to investigate the effectiveness of socket designs and to understand differences between them.

State-of-the-art prosthetic sockets are designed and hand-crafted individually. Depending on the socket concept, a Plaster of Paris (POP) wrap cast is manually applied over the residual limb (residuum) or over the elastomeric liner covering the residual limb with the aim to capture a modified shape of the soft tissues. Prosthetists shape the POP during casting for the PTB socket using his/her hands (Hands-on) while in the ICECast a pressure bladder is used for this purpose (Hands-off). This shape is used to produce a positive model, which is afterwards adapted (rectified) according to one of the number of design paradigms. These procedures are highly individual, often inconsistent, and based on tacit knowledge. The performance by an individual prosthetist will be strongly influenced by personal experience, skill, and beliefs [12, 13]. When the socket manufacturing process is not reproducible it will, besides the obvious prosthetic fit issues, affect the positioning of the socket relative to the prosthetic foot (alignment) and hence alter ambulation. Without doubt those difficulties compromise the prosthetic rehabilitation process [13, 14].

The shape capturing consistency of Hands-on and Hands-off sockets has been compared using a manikin model [13]. The Hands-off concept showed a constant pattern of maximum radius variation of 1.4 mm, whereas the Hands-on concept had maximum radius variations of approximately 2.4 mm and 5 mm in the distal part and proximal part of the model, respectively. Quantification of inter- and intra-socket shape and volume differences requires accurate alignment of a three-dimensional (3-D) model of the residual limb in a common coordinate system.

In surface scanning methods, the morphological information about the bone and its relation to the surface of the socket, which could be useful in better understanding the socket fit, is missing. The Spiral X-ray Computer Tomography (SXCT), MRI, and Ultrasound provide both internal and external limb information. Therefore, the rigid internal limb structure (e.g., tibia) can be used as a reference to align multiple 3-D models of a residual limb [15, 16]. These methods can also be used to scan the residual limb while the socket/prosthesis is donned. Smith et al. obtained two SXCT scans of seven transtibial residual limbs on each of two sessions. The tibia was segmented from SXCT scans and then used to register all inter- and intrasession scans to a common coordinate system. The results of their study showed that this technique of registration has an error of approximately 1% relative to the mean volume of the residual limb [16].

MRI is a nonionising high resolution imaging technique which can provide a clear distinction between tissues. Studies have shown that MRI is an accurate method of soft tissue and bone dimension and volume measurement and has been used to estimate accurate morphological information of different tissues, for example, bone, muscle, and articular cartilage [17–19]. Additionally, the use of MRI in a residual limb morphological measurement, when common casting materials were used, was validated in previous experiments [15, 20, 21].

The aim of this study was to examine Hands-off and Hands-on inter- and intracast consistency in the form of residual limb shape, volume, length, and transverse cross-sectional surface area and circularity using MRI.

## 2. Methods

Twelve amputees with an established residual limb (at least six months of using prosthesis) without blisters and other skin problems were recruited. The Ethical approval was granted by NHS Glasgow Ethics Committee (reference no. SN08NE446) and all amputees gave informed consent before participation.

The residual limb was cast four times in a single session sequence, twice for Hands-on and twice for Hands-off method, by a single certified prosthetist with over 30 years of experience. A random selection sequence was adopted to minimise the effect of one cast on the volume of the residual limb for the subsequent cast. The wet POP, used for casting, was doped with 1 gr/lit Copper Sulphate (CS) to enhance signal intensity for improved image segmentation in the MRI scan. The plaster cast, in both casting methods, was extended over the femoral condyles to minimise the cast-residual limb movement. In addition, due to subcutaneous fat causing a chemical shift artefact in the MRI scan, eight layers of Perlon stockinet were applied between residual limb and the overlaying POP (in Hands-on) or silicone liner (in Hands-off) to create a gap (*≈*3 mm) to improve image segmentation of the residual limb skin and the casting material.

After each cast the residual limb was scanned using MRI. In order to prevent image distortion resulting from limb movement, the patella was rested over a knee cap receptacle made from polyethylene foam and the thigh region was fixed using pads and straps. The sagittal Fast Spoiled Gradient Recall Echo (FSPGR) pulse sequence with the following parameters was adopted: field intensity 3 T, repetition time 6.9 s, time of echo 1.5 s, inversion time 500 ms, Bandwidth 31.25 KHz, flip angle 12 deg, matrix 256 × 256, slice thickness 1.2 mm, voxel dimensions 1.17 × 1.17 × 0.6 mm, and a 1-signal average.

**Figure 1:**
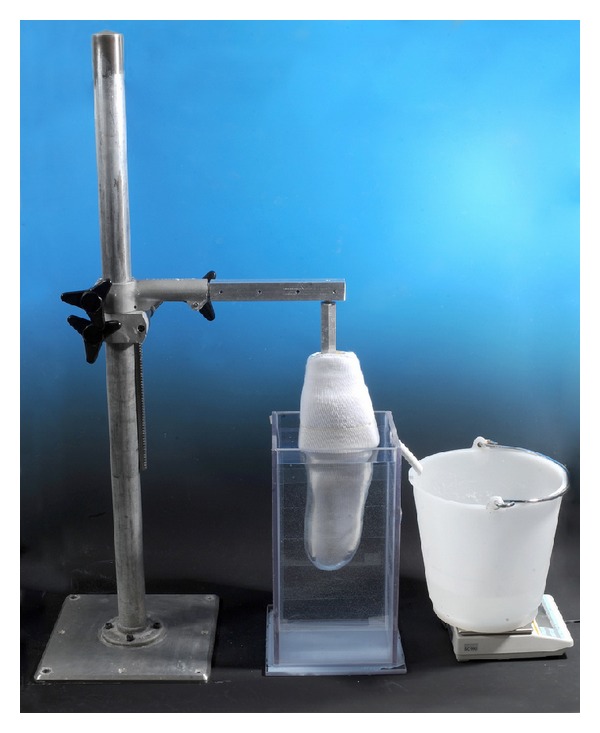
Water weighting of residual limb covered with one layer of Terry Cloth sock.

Amputees usually add or remove socks over residual limb to compensate for residual limb volume fluctuation. Based on amputees' experience, Lilja and Öberg [22] assumed the bad fit criteria to be one or two layers of socks over the residual limb, that is, the use one or two socks by the amputee; then the new socket was made. Therefore, In order to interpret the shape and volume results in a clinical meaningful way the percentage volume of one layer of a Terry Cloth Sock (Otto Bock) over a residual limb model was measured using the water displacement method, Figure 1. This was ((1765.1−1635.2)/1635.2) × 100 = 7.943%. The sock was 2.28 mm thick measured using a dial thickness gauge (accuracy of 0.025 mm) before pulling over the residual limb model.

After removing the subject identifiers, MRI data was exported to the Analyze 0.9 software and the voxel size was modified to an isotopic cubic shape (*x* = *z* = *y* = 0.6 mm). Soft tissue and bone were segmented semiautomatically from surrounding materials, for example, silicone and POP. The accuracy of the segmentation procedure has been previously reported using animal specimen and was 0.43% for surface area and 2.25% for volume measurement [20].

**Figure 2:**
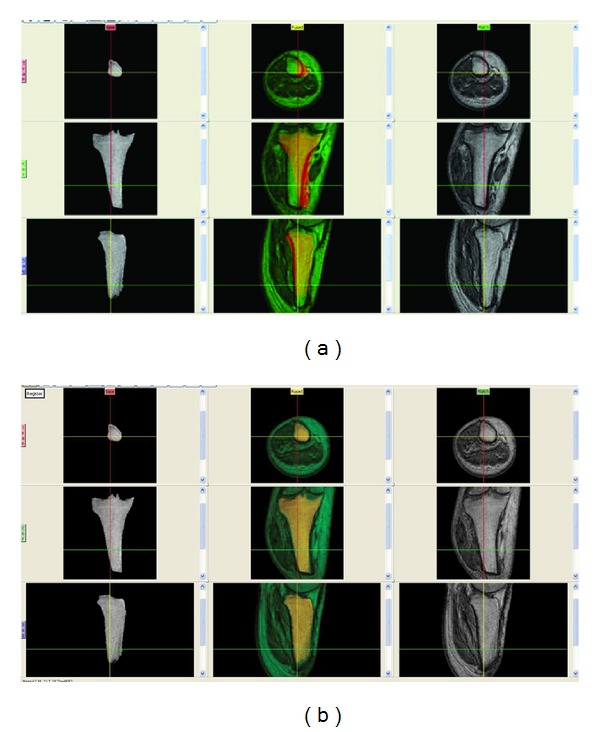
Volume registration in relation to the aligned tibia bone. (a) Before registration; (b) after registration.

To allow spatially registration of several volume images of the same residual limb one of the volume images was selected randomly and then the tibia bone was also semi-automatically segmented. The tibia was then aligned so that the transverse image slices were parallel to the proximal surface of the bone. Finally, all other volume images were spatially registered to the aligned segmented tibia bone using a Normalised Mutual Information algorithm which allows the precise alignment of 3-D data to be achieved, (Figure 2).

**Figure 3:**
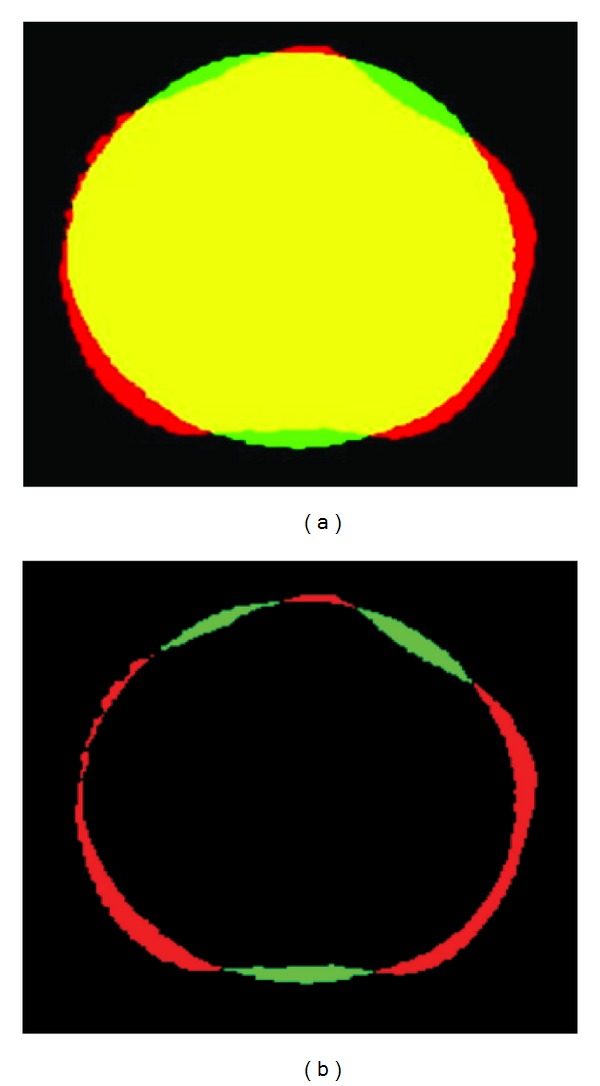
Shape difference between Hands-off and Hands-on. (a) Superimposed slices of two scans; (b) absolute shape difference. Yellow, red, and green regions are the common points, Hands-on, and Hands-off, respectively.

After registration and to standardise the volume scans, all slices above the 30th slice proximal to the tibial plateau were removed in all volume data. This enabled a consistency in anatomical residual limb length for subsequent comparison. Then, all data were reformatted into the binary format for the purpose of automatic shape and volume calculation using the software. The binary data was used to measure the absolute shape difference of a pair of volume images and in constructing colour coded images, Figure 3.

The transverse cross-sectional surface area (CSSA) and cross-sectional circularity (CSC) (Circularity is a dimensionless value. It was measured as the ratio of the perimeter squared of the region to the area of the region (*P* ^2^/*A*)) of residual limb in all slices of all volume images were also automatically calculated by the software. Additionally, the lengths of all four scans were calculated as the number of transverse slices in which the residual limb appeared multiplied by the slice thickness (0.6 mm).

Residual limb volumes of all images were also measured. Then each volume image was sectioned into four regions of antrolateral (AL), anteromedial (AM), posetrolateral (PL) and posteromedial (PM) by defining two sagittal and coronal cutting planes. The sagittal cutting plane was defined as passing through the intercondylar tubercles of the tibia and the coronal cutting plane passing through the midpoint of the tibia plateau. Additionally volume images were sectioned into three regions (distal, middle, and proximal) using two transverse cutting planes, located at one-third and two-thirds the averaged length of the residual limb. Lastly the overall and regional absolute shape differences were calculated. For CSSA and CSC data, three slices were chosen randomly in each of proximal, middle, and distal regions of each cast, for the purpose of statistical analysis.

Intraclass Correlation Coefficient (ICC) and the Coefficient of Variation (COV) [23] were used to measure the consistency of each casting concept (i.e., Hands-on and Hands-off). ICC is the measure of reliability of the ratings. An ICC value greater than 0.7 is regarded as acceptable. The COV is the standard deviation divided by the mean and is used to show the amount of deviation as a percentage of the mean. A limitation is the sensitivity of COV when the mean value is near zero. The COV of less than 5% is judged to be as acceptable repeatability. The paired *t*-test was used to assess the statistical significance difference between the two casting methods. The Shapiro-Wilks test was used to see if the distribution of the values differed significantly from a normal distribution. When the normal distribution could not be justified the paired Wilcoxon test was used. Bland and Altman (BA) plots were used to highlight the mean difference and the variability of the two measurements [24].

## 3. Results

### 3.1. Transverse Cross Sectional Surface Area and Circularity Difference

**Figure 4:**
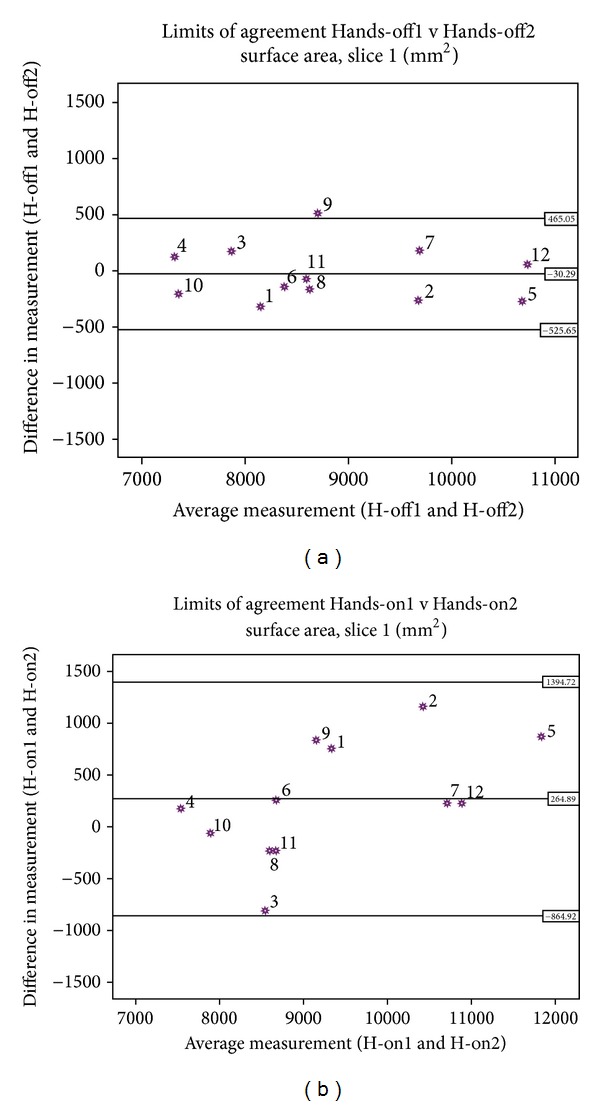
Bland and Altman plot for intracast CSSA of both Hands-off (a) and Hands-on (b) castings in slice 1.

The Hands-on method resulted in a larger intra cast CSSA mean difference than the Hands-off method (Tables 1 and 2). It was noticed from the tables and the BA plots that the proximal region showed a larger CSSA intra cast mean difference and variability in the Hands-on casting and a larger intercast variability. For presentation, the BA plot for intra cast CSSA of both casting methods for slice 1 is presented in Figure 4. At the far distal region (slice 9), a larger inter- and intra cast CSC mean difference and variability was observed in both casting methods. Additionally, the intercast CSSA and CSC mean difference and variability were larger than that of either Hands-on or Hands-off intra cast results.

Neither Hands-on nor Hands-off intra cast CSSA and CSC differences were statistically significant, with the exception of the Hands-on CSC of the first slice. There were, however, statistically significant differences between the Hands-on and Hands-off in CSSA at the far distal region (slice 9) and in CSC in the proximal region (slice 2) (Tables 1 and 2).

### 3.2. Length Difference

**Figure 5:**
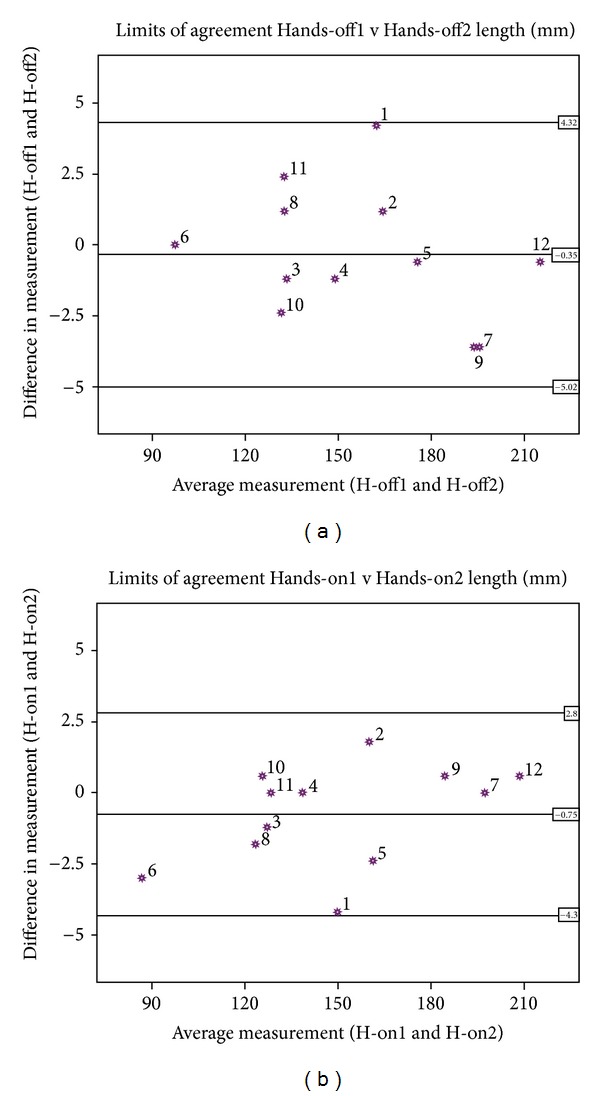
Bland and Altman plot for intra cast length of both Hands-off (a) and Hands-on (b) castings.

The ICC value of more than 0.7 is regarded as repeatable [25]. The intra cast length difference is minimal but the intercast length difference is noticeable, (Table 3). The ICC results show that both Hands-off and Hands-on concepts are repeatable for residual limb length, (Table 3). The residual limb length difference of Hands-off and Hands-on were not statically significant (mean difference = 7.6 mm, SD = 4.315, and *P* = 0.595). However, greater intra cast length variability in the Hands-off method than the Hands-on casting was noticed (Table 3) (Figure 5).

### 3.3. Volume Difference

The ICC results reveal that the overall volume readings of both Hands-off and Hands-on concepts are repeatable, (Table 3). Additionally, there was no significant difference between Hands-off and Hands-on overall volume measurements (mean difference = 23462.04 mm^3^, SD = 29734.80, *P* = 0.872). However, the intra cast volume difference of Hands-off casting method was less than that of Hands-on method with less variability, (Table 3).

Furthermore, the ICC test showed that both casting methods resulted in a repeatable intra cast regional volume measurement (Table 4). Although not statically significant, the Hands-on intra cast mean volume difference and variability were larger than the Hands-off results in AL, AM, PL, PM, and the proximal regions. For the middle and distal regions the Hands-off method showed larger intra cast mean difference and variability. The intercast volume difference was not significant in any region of the residual limb (*P* > 0.05) (Table 5).

**Figure 6:**
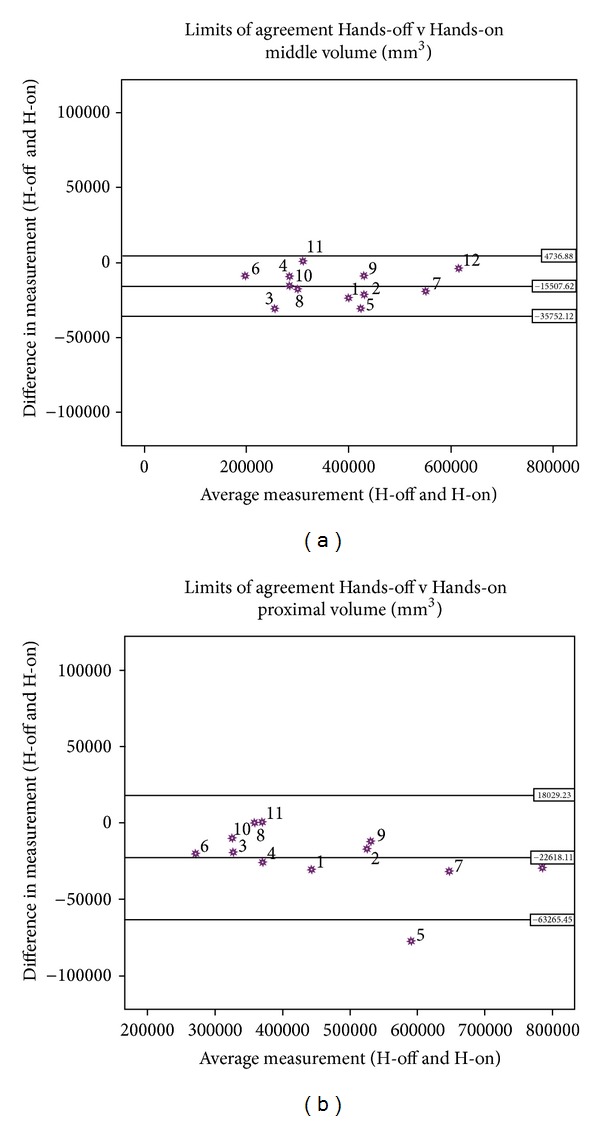
Bland and Altman plot for intercast volume of middle (a) and proximal (b) regions.

The Hands-on intra cast volume variability was larger at the PM region than the other three regions (AL, AM, and PL). For the Hand-off method, there was larger intra cast volume variability at the posterior region compared to the anterior region (Table 4). This region also showed a larger intercast volume variability (Table 5). The Hands-on method showed a greater volume variability at the proximal region compared to the middle and distal regions, whereas the Hands-off method showed a greater volume variability at the middle region (Table 4). The middle region showed less intercast volume variability compared to that of proximal and distal regions, (Table 5), (Figure 6).

### 3.4. Shape Difference

A CoV of less than 5% is judged to be acceptable [23]. The results show that both casting methods have large intra cast overall shape CoV values. However, the intra cast shape consistency is slightly larger for Hands-on method than Hands-off method (CoV Hands-on = 49.68% and CoV Hands-off = 61.97%) but the mean shape difference is higher (Hands-off mean difference (SD) = 53523.24 (33169.73) mm^3^ and Hands-on mean difference (SD) = 90464.92 (44964.24) mm^3^).

Both casting methods showed large regional CoV values. Compared to the Hands-off casting, the Hands-on method resulted in smaller CoV values in all seven regions but had a larger mean shape difference (Table 6). The posterior region of the residual limb, in both casting methods, has larger mean shape difference than the anterior region. Additionally the PM region has the highest shape CoV in both methods, hence less shape consistency. The AM region and AL resulted in the smallest CoV in Hands-on and Hands-off castings, respectively. The middle region of the residual shows the maximum CoV in both casting method. However the proximal region shows the larger mean shape difference than the distal and middle regions in the Hands-on concept. In Hands-off concept the distal region has the highest mean shape difference.

### 3.5. Clinical Significance of the Results

The shape and volume differences were tested against the percentage volume of one layer of Terry Cloth sock over the residual limb. First, the percentage volume of one layer of sock (7.94%) was subtracted from one of the repetitions (i.e., 92.06% of original volume) and then difference between this value and the second repetition was tested using the *t*-test. The results show that the 92.06% volume of one repetition was significantly different (*P* < 0.05) from the second repetition in both casting methods. (Table 7). In other words, the intra cast differences were less than the clinical meaningful volume fluctuation of the residual limb (i.e., 7.94%). In addition, 92.06% of average volume of Hands-off casting repetitions was significantly different from average volume of Hands-on casting repetitions (*P* < 0.05).

The amount of intra cast absolute shape difference was given in cubic millimetres. Therefore, the 7.94% of average volume of four repetitions (of both casting concepts) were calculated and compared to the intra cast shape difference of either casting concept using *t*-test. The difference between volume of one layer of sock and the Hands-off shape difference was significant (mean difference (SD) = −26454.85 (17865.87) mm^3^, *P* = 0.001). In other words it was less than the volume of one layer of sock. The Hands-on shape difference was not significantly different from the volume of a Terry Cloth sock (mean difference (SD) = 10486.82 (41532.30) mm^3^, *P* = 0.400), that is, equal to the volume of one layer of sock.

## 4. Discussion

The quality of prosthetic socket fit is influenced by consistency in shape capture process. In this study twelve residual limbs were casted using two common casting methods, that is, Hands-on and Hands-off. Then the CSSA, CSC, length, volume, and shape of residual limb were measured using MRI and then compared for inter- and intra cast consistency of methods.

In the Hands-on method, following the POP application, a prosthetist manually applies a pressure over the residual limb to preshape the cast. This approach may result in an inconsistent outcome but is influenced by prosthetist skill and dexterity. This is likely to be the reason for the large inter- and intra cast inconsistency of the measured variables for the Hands-on method at the proximal region of the residual limb. Similar findings in relation to manual dexterity were reported by Buis et al. [13]. In their study, the Hands-off concept showed a constant pattern of maximum radius variation of 1.4 mm, whereas the Hands-on concept had a maximum radius variation of approximately 2.4 mm and 5 mm in the middle and proximal part of the residual limb model, respectively. The results of their study are in agreement with the result of this study as the CSSA variability in Hands-off casting shows a constant pattern with a smaller mean CSSA throughout the length of the residual limb.

Although there was no statistical significant intercast CSSA difference, except at the far distal part (slice 9) of the residual limb, the Hands-off CSSA mean and intra cast variability were smaller than those of the Hands-on method (Table 1). This could be due to the uniform pressure around the residual limb produced when using the air bladder. In the study by Kahle [26], the percentage diameter relative to the residual limb was +19.4% and +4.4% for AP diameter at the tibial tuberosity level for HS and PTB sockets, respectively, and mediolateral percentage differences were HS = +6.3% and PTB = +3.7%. Kristinsson reasoned that the combination of the radial pressure around the residual limb and the effect of the silicon liner on downward displacement of the skin results in elongation of the residual limb soft tissue [4]. The uniform force application around the residual limb and the distal traction of the soft tissue in the Hands-off casting could result in longer residual limb and, if the volume is unchanged, smaller CSSA. The distal end pressure could help in blood return and prevent oedema.

Both investigated casting concepts showed intra cast length consistency with high ICC values. However, the Hands-off method showed less intra cast length mean difference and variability. Although not statically significant, the Hands-off method resulted in a longer cast length (mean difference = 7.6 mm). Similar results were reported by Kahle et al. [26] where the HS socket was longer than the PTB socket. Length percentage differences, relative to the length of the residual limb, were 20.1% and −3.8% for HS and PTB socket, respectively [26].

There was a statically significant intra cast CSC difference in the far proximal region (slice 1) in Hands-on method and in the intercast CSC at the proximal level (slice 2). This could be a reason for inconsistency of the results at the proximal region due to manual dexterity in the Hands-on method. Both casting method resulted in a large CSC mean and SD in the distal region possibly due to the loose end soft tissue. Although not statistically significant, the CSC values are slightly smaller for the Hands-off method, hence more circular cross-section, than the Hands-on. This could be the result of mechanical compliance of soft tissue subjected to the uniform pressure applied by the casting air bladder.

In the study by Yiğiter et al. [27], using water filling method, the PTB socket compared to the Total Surface Bearing (TSB) socket resulted in a larger volume size (PTB = 772.2 ± 238.2 cm^3^, TSB = 600.0 ± 182.8 cm^3^). In our study the Hands-on mean volume was 23462.04 mm^3^ larger than that of Hands-off method. However, the percentage volume difference in our study was smaller, 2.35% compared to 22.29% in Yiğiter study. Yiğiter measured the volume of sockets, whereas in this study the volume of POP cast (the shape capturing process) was measured. Following the shape capturing, the socket is made after the process of cast rectification which includes adding or removing plaster on the positive plaster cast of residual limb in the PTB concept. This may add to the further volume difference of the Hands-on and Hands-off sockets.

In this study, the posterior region of the residual limb showed greater intra- and intercast volume variability. Additionally, greater intra cast shape mean difference and variability were observed in this region irrespective of casting method. Furthermore, the Hands-off method resulted in larger shape and volume mean difference and variability mostly in the middle and distal regions, whereas the Hands-on method showed greater shape and volume mean difference and variability at the proximal region. The inconsistency in the middle region of the residual limb could be explained by soft tissue deformation during casting. The soft tissue displacement over underlying bone is greater in areas with larger amount soft tissue mass [28]. The POP is manually wrapped around the residual limb, resulting in deformation and displacement of the soft tissue in relation to underlying bone. In the case of Hands-off method, the soft tissue could be slightly displaced, mainly in the areas with significant amount of soft tissue, prior to air bladder application. In the study described by Buis et al. where a manikin model was used it was reported that the greatest variation of the used Hands-on method was found to be at the posterior region and the greater variation for Hands-off concept was mostly located in the lateral region [13].

Neither Hands-on nor Hands-off method has acceptable shape consistency (the CoV was 49.68% and 61.97% for Hands-on and Hands-off concept, resp.). The Hands-on method resulted in a larger intra cast shape mean difference but smaller SD relative to the mean difference value compared to the Hands-off method. The shape consistency of the Hands-off casting method could depend on factors such as consistent bladder pressure setting during casting and recasting, direction of proximally applied force to the bladder by prosthetist and time delayed after POP application and use of bladder over the residual limb. As opposed to the volume comparison, the intercast shape comparison was not possible due to the lack of the intra cast shape consistency. Therefore, calculating the average shape of the two repetitions was not logical as none of repetitions could be assumed to be a true value.

In some cases the residual limb was longer and slimmer in one repetition of the Hands-off concept than the other. If the amount of the equal pressure over the residual limb applied by the bladder increases, having the overall volume of the residual limb constant, the transverse cross sectional surface area will decrease and the length will increase. Additionally, in the Hands-off casting the bladder is attached to the distal pin of the silicone liner and the liner is in close contact with the skin. Any change in the direction of the proximally applied force to the bladder would result in a slight change in direction of the force applied over the residual limb. Furthermore, in the first repetition of Hand-off casting for the second amputee, the bladder was unintentionally used following considerable time elapse after the POP application. In this case the POP was partially cured. It was noted that the resulted residual limb shape was not the same as the other repetition in circularity. The shape formation of a semirigid POP cast under a uniform pressure would not necessarily be equal throughout the entire medium.

In order to identify the proper time for the permanent prosthetic fitting, Lilja and Öberg [22] measured postamputation volume fluctuation of the residual limb using laser scanning. Based on amputees' experience, they assumed the bad fit criteria as to be one or two layers of socks over the residual limb, that is, using one or two socks by the amputee; then the new socket must be made. They measured the percentage volume of the one and two socks over the residual limb as to be 5.2% and 9.4% [22]. Their results of sock volume percentage is in agreement with that of Fernie and Holliday [29]. Also Sanders et al. calculated that the uniform volume change of 5% in a limb with 90 mm diameter would be 1 mm change in diameter [30]. The percentage volume difference of one layer of the Terry Cloth sock was measured using water displacement technique. This was 7.94% for a sock thickness of 2.28 mm. The difference between this study and those of Lilja and Sanders is possibly due to the thickness of the sock used.

**Figure 7:**
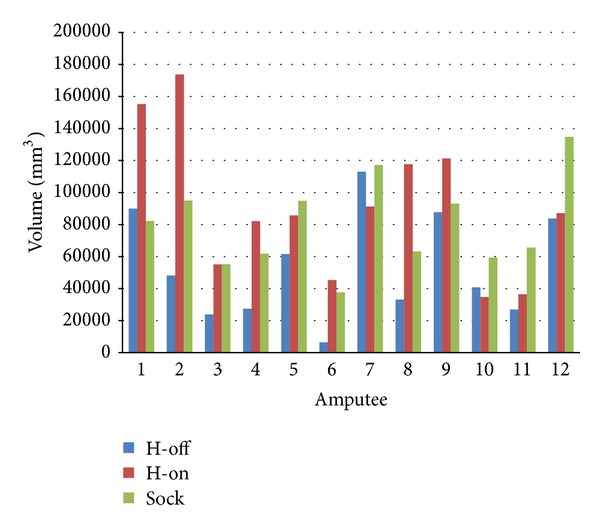
Stock volume, Hands-off, and Hands-on shape difference.

The results show that neither intra cast nor the intercast volume differences are clinically significant, that is, the amount of inter- and intra volume difference is less than the volume of one Terry Cloth sock over the residual limb. The result of statistical test and the graph (Figure 7) show, that the Hands-off intra cast shape difference is less than 7.94% of total residual limb volume, whereas there is no significant difference between the Hands-on shape difference and 7.94% of total residual limb volume. It is worth noting that this results show the intra cast shape difference of shape capturing process. However, the mean difference and variability of the intercast surface area and circularity are larger than that of either Hands-off or Hands-on intra cast results. This was expected as the Hands-on casting has a different approach in shaping the residual limb than the Hands-off method. Therefore, the intercast shape difference could possibly be larger than the volume of one layer of sock over the residual limb.

In the Hands-off casting method an air bladder based casting device (ICECast compact) is used to apply an equal pressure around the residual limb during casting. When a uniform force is applied to the soft tissue it responds with the same amount of force. If the tissue is assumed incompressible and it does not escape under the load, the soft tissue shape would be would be a result of the mechanical compliance of the soft tissue. The residual limb soft tissue consists of several layers of different properties, each responding differently under load. The force flow chooses the stiffest path as the stiffer tissue takes charge [31]. Additionally, the shape of underlying rigid structures, that is, bone, in combination with the overlying soft tissue thickness is playing roll in defining the final shape of the residual limb under a uniform pressure. Therefore, the shape of the socket is dictated by the shape and mechanical property of the residual limb. Each element of the limb contributes in weight bearing proportionally to its mechanical property. This could be a possible approach to achieve a total surface bearing socket. Having the properties of the soft tissue unchanged during repeated casting and under the same amount of a uniform pressure, applied by the bladder, the residual limb shape is expected to be consistent in a repeated casting. This could be a reason for the less intra cast shape and volume differences in the Hands-off casting method and showing no clinical significant shape inconsistency.

## 5. Conclusion

The residual limb shape capturing consistency, as the first stage of socket manufacturing process, is a first step to evaluate effectiveness of socket designs and to understand differences between them. The results show that both casting method, have intra cast volume consistency and there is no significant volume difference between two methods. Additionally, inter- and intra cast mean volume difference was not clinically significant based on the volume of one sock criteria.

The inconsistent results of the Hands-on method were expected because of hand dexterity in casting. The Hand-off method, relative to the Hands-on method, resulted in consistent results. However, this relies on factors such as meticulously setting and maintaining the bladder pressure and the proximally applied force to the bladder by prosthetist. A special designed casting device (e.g., automatic air pressure setting feature) could minimise the effect of these factors, especially in areas of residual limb with large amount soft tissue. Providing these factors, the Hands-off method has a potential to result in an even more consistent socket through an objective socket manufacturing procedure. Therefore, not only this could improve amputees' experience but also provide possibility to better understand socket designs and investigate other factors influencing prosthesis function such as rectification and alignment.

It is suggested for later studies that inter- and intra rater consistency of the casting could be examined. The same approach can be attained to evaluate inter- and intra shape and volume of cast rectification in different socket concepts as well as other stages of prosthesis fabrication such as rectification and/or alignment. Furthermore, other pressure casting devices, such as the hydrocast method, can be utilised to evaluate the shape and volume consistency of the pressure casting method.

The quality of socket fit relies on the socket-residual limb coupling stiffness providing comfort with no pain or tissue damage. Therefore, it is recommended to consider “amputee's comfort” in any socket/prosthesis investigation. When objective assessment of socket geometry, soft tissue characteristics, prosthetic components, and so forth, is combined with amputees' subjective feedback, then the better understanding of the socket designs would be possible.

## Figures and Tables

**Table 1:** The CSSA mean (SD) of each repetition of Hands-off and Hands-on casting methods and average of two repetitions, CSSA intracast absolute mean difference, and the paired *t*-test significance of intra- and intercast CSSA difference.

Region	Slice	Hands-off (mm^2^)				Hands-on (mm^2^)				Hands-off V Hands-on		
		Rep 1	Rep 2	Absolute intra cast difference	Hands-off intra cast significance (*P* < 0.05)	Rep 1	Rep 2	Absolute intra cast difference	Hands-on intra cast significance (*P* < 0.05)	Average Hands-off	Average Hands-on	Inter cast significance (*P* < 0.05)
	1	8799.0 (1150.1)	8829.3 (1170.8)	207.4 (124.1)	0.680	9484.5 (1489.1)	9219.6 (1186.5)	487.0 (370.1)	0.133	8814.2 (1153.9)	9352.0 (1316.4)	0.299
Proximal	2	8605.0 (1134.3)	8656.3 (1168.0)	194.9 (147.3)	0.484	9006.1 (1420.6)	8845.4 (1174.7)	376.7 (338.6)	0.281	8630.7 (1144.7)	8925.8 (1280.1)	0.558
	3	8319.5 (1075.4)	8342.6 (1087.5)	151.6 (141.7)	0.712	8619.7 (1360.1)	8607.4 (1169.3)	280.7 (240.9)	0.913	8331.0 1076.3	8613.5 (1254.1)	0.560

	4	7530.9 (1241.3)	7567.0 (1243.0)	150.3 (149.4)	0.570	7879.9 (1276.3)	7860.9 (1217.9)	206.4 (170.5)	0.814	7548.9 (1237.6)	7870.4 (1239.9)	0.532
Middle	5	6940.7 (1428.1)	6963.8 (1369.4)	195.9 (218.2)	0.793	7242.2 (1448.0)	7209.0 (1412.9)	233.7 (159.7)	0.699	6952.2 (1391.1)	7225.6 (1423.2)	0.639
	6	6497.2 (1254.3)	6508.9 (1161.8)	206.2 (243.5)	0.903	6772.8 (1294.5)	6756.8 (1215.2)	251.9 (177.5)	0.865	6503.1 (1198.0)	6764.8 (1245.5)	0.605

	7	5042.8 (1306.1)	5058.3 (1201.7)	195.3 (222.0)	0.861	5126.4 (1743.3)	5133.5 (1533.8)	302.9 (274.8)	0.952	5050.5 (1245.9)	5129.9 (1629.6)	0.895
Distal	8	3504.8 (1328.0)	3528.9 (1180.5)	207.5 (178.1)	0.771	3229.5 (1860.1)	3276.2 (1610.9)	315.2 (274.8)	0.711	3516.8 (1248.6)	3252.9 (1726.9)	0.273^*≠*^
	9	2082.8 (1428.9)	2213.5 (1277.3)	263.0 (298.5)	0.261	1380.9 (1898.8)	1465.1 (1604.4)	276.3 (274.3)	0.468	2148.1 (1341.7)	1423.0 (1747.0)	0.021^*≠*^

^≠^Normality was not assumed.

**Table 2:** The CSC mean (SD) of each repetition of Hands-off and Hands-on casting methods and average of two repetitions, CSC intracast absolute mean difference, and the paired *t*-test significance of intra- and intercast CSC difference.

Region	Slice	Hands-off				Hands-on				Hands-off V Hands-on		
		Rep 1	Rep 2	Absolute intra cast difference	Hands-off intra cast significance (*P* < 0.05)	Rep 1	Rep 2	Absolute intra cast difference	Hands-on intra cast significance (*P* < 0.05)	Average Hands-off	Average Hands-on	Inter cast significance (*P* < 0.05)
	1	1.16 (0.03)	1.16 (0.02)	0.03 (0.03)	0.881	1.18 (0.02)	1.20 (0.04)	0.02 (0.02)	0.004	1.16 (0.01)	1.20 (0.03)	NA
Proximal	2	1.16 (0.03)	1.16 (0.02)	0.02 (0.02)	0.600	1.19 (0.03)	1.20 (0.02)	0.02 (0.02)	0.104	1.16 (0.02)	1.20 (0.03)	0.004
	3	1.18 (0.05)	1.16 (0.02)	0.04 (0.03)	0.225	1.19 (0.05)	1.20 (0.04)	0.03 (0.02)	0.336	1.17 (0.03)	1.19 (0.04)	0.204^*≠*^

	4	1.17 (0.04)	1.16 (0.02)	0.03 (0.02)	0.256	1.18 (0.03)	1.19 (0.04)	0.03 (0.03)	0.830	1.17 (0.03)	1.18 (0.03)	0.083^*≠*^
Middle	5	1.18 (0.04)	1.16 (0.02)	0.03 (0.02)	0.272	1.18 (0.03)	1.18 (0.04)	0.01 (0.01)	0.624	1.17 (0.03)	1.18 (0.03)	0.431
	6	1.17 (0.03)	1.17 (0.02)	0.03 (0.02)	0.820	1.18 (0.02)	1.19 (0.04)	0.03 (0.03)	0.325	1.17 (0.02)	1.19 (0.03)	0.072

	7	1.19 (0.04)	1.19 (0.04)	0.04 (0.02)	0.887	1.20 (0.03)	1.20 (0.04)	0.03 (0.02)	0.739	1.19 (0.03)	1.20 (0.03)	0.497
Distal	8	1.20 (0.04)	1.21 (0.03)	0.04 (0.03)	0.584	1.20 (0.04)	1.22 (0.06)	0.04 (0.04)	0.381	1.21 (0.03)	1.21 (0.04)	0.794^*≠*^
	9	1.32 (0.33)	1.22 (0.04)	0.10 (0.30)	0.290	1.34 (0.17)	1.33 (0.15)	0.11 (0.15)	0.867	1.27 (0.18)	1.33 (0.13)	0.650

^≠^Normality was not assumed.

**Table 3:** Mean, standard deviation of the residual limb length and volume for each repetition of casting concepts, intra cast mean difference, and the ICC value.

Region	Mean (SD) mm^3^		Mean difference (SD) mm^3^	ICC	Mean (SD) mm^3^		Mean difference (SD) mm^3^	ICC
	H-off1	H-off2	(H-off1 and H-off2)		H-on1	H-on2	(H-on1 and H-on2)	
Length (mm)	156.70 (33.63)	157.05 (34.36)	−0.35 (2.34)	0.998	148.90 (35.42)	149.65 (34.37)	−0.75 (1.77)	0.999

Volume (mm^3^)	993910.27 (350350.74)	997189.23 (343389.39)	−3278.95 (26771.71)	0.997	1025034.61 (372591.91)	1012988.99 (350130.56)	12045.62 (48515.49)	0.991

**Table 4:** The regional mean and standard deviation of volume (mm^3^) for each repetition (Rep 1 and Rep 2) of casting concepts, the ICC, and intra cast volume difference.

Region	Mean (SD) mm^3^		Mean difference (SD) mm^3^	ICC	Mean (SD) mm^3^		Mean difference (SD) mm^3^	ICC
	H-off1	H-off2	(H-off1 and H-off2)		H-on1	H-on2	(H-on1 and H-on2)	
Anterolateral	285567.53 (144163.88)	284904.17 (143181.02)	663.35 (4466.00)	1.000	287513.47 (140635.44)	275816.08 (135721.14)	11697.39 (18394.33)	0.988
Anteromedial	184602.26 (61934.75)	184430.92 (62131.10)	171.34 (5135.96)	0.997	191006.50 (64020.38)	1803092.77 (58299.46)	7913.73 (10871.89)	0.977
Posterolateral	312730.60 (115808.00)	314824.59 (113839.87)	−2093.99 (11326.17)	0.995	319797.14 (127496.42)	327316.82 (125287.45)	−7519.68 (13678.61)	0.993
Posteromedial	221712.65 (60022.84)	221641.40 (59877.60)	71.25 (15103.64)	0.971	236744.99 (66843.42)	235752.47 (59361.07)	992.52 (24387.24)	0.931
Distal	190600.10 (64744.37)	191701.39 (63435.98)	−1101.28 (11641.72)	0.948	176594.38 (73801.56)	176779.01 (65441.12)	−184.63 (16398.34)	0.975
Middle	365797.29 (126577.84)	364928.64 (122581.30)	2804.93 (14301.54)	0.995	382273.05 (127010.40)	379468.12 (121486.35)	868.64 (13325.32)	0.994
Proximal	449826.34 (150318.7)	450846.36 (151331.43)	−1020.02 (11601.41)	0.997	477764.39 (164951.10)	468144.53 (156290.14)	9619.86 (21634.93)	0.990

**Table 5:** Mean and standard deviation of regional volume for Hands-on, Hands-off casting concepts and inter cast volume difference, significance of inter cast regional volume difference, *t*-test.

Region	Mean (SD) mm^3^		Mean difference SD mm^3^	Significance (0.05)
	H-off	H-on	(H-off and H-on)	
Antero-lateral	285235.85 (143655.94)	281664.77 (137893.76)	3571.07 (12395.41)	0.951
Antero-medial	61979.83 (17892.036)	60984.99 (17604.85)	2533.05 (7190.39)	0.921
Postero-lateral	313777.59 (114589.20)	323556.98 (126211.59)	−9779.39 (16909.91)	0.844
Postero-medial	59472.72 (17168.29)	62025.84 (17905.32)	−14571.71 (15724.53)	0.563
Distal	191150.74 (63828.64)	176686.69 (69263.14)	14464.05 (20596.03)	0.600
Middle	365362.97 (124417.33)	380870.60 (124073.179)	−15507.62 (101122.25)	0.763
Proximal	450336.35 (150714.33)	472954.46 (160314.44)	−22618.11 (20323.67)	0.725

**Table 6:** Mean, standard deviation, and CoV (%) for regional intra cast shape difference of Hands-off and Hands-on methods.

Region	Mean (SD) mm^3^		CoV	
	H-off	H-on	H-off	H-on
Anterolateral	13262.40 (8748.46)	23305.66 (13229.65)	65.960	56.770
Anteromedial	8906.41 (6400.15)	16092.95 (8621.07)	71.860	53.570
Posterolateral	16355.30 (10419.91)	25402.99 (14007.36)	63.710	55.140
Posteromedial	14341.32 (10869.77)	25661.52 (17497.69)	75.790	68.190
Distal	19870.41 (13333.14)	25574.58 (14187.62)	67.100	55.480
Middle	15290.86 (10851.94)	30938.05 (19594.40)	70.970	63.330
Proximal	17604.79 (10146.99)	33950.67 (14858.50)	57.640	43.770

**Table 7:** Mean, standard deviation, and significance of one layer of sock.

Volume difference	Mean	SD	Significance (*P* < 0.05)
(0.92 × H-off1)-H-off2	−82195.43	32406.75	<0.001
(0.92 × H-on1)-H-on2	−69342.12	41870.52	<0.001
(0.92 × H-off)-H-on	−102508.69	48609.5	<0.001

## Abbreviations

3-D: Three-dimensional
AL: Anterolateral
AM: Anteromedial
BA: Bland and Altman
CoV: Coefficient of Variation
CSC: Cross-sectional Circularity
CSSA: Cross-sectional Surface Area
CS: Copper Sulphate
FSPGR: Fast Spoiled Gradient Recall Echo
HS: Hydrostatic Socket
ICC: Intraclass Correlation Coefficient
MRI: Magnetic Resonance Imaging
PL: Posterolateral
PM: Posteromedial
POP: Plaster of Paris
PTB: Patellar Tendon Bearing
SD: Standard Deviation
SXCT: Spiral X-ray Computer Tomography
TSB: Total Surface Bearing.
